# A Comparison of Nailfold Video Capillaroscopy Findings in Mixed Connective Tissue Disease Interstitial Lung Disease vs Systemic Sclerosis Interstitial Lung Disease: A Single-Centre Study

**DOI:** 10.31138/mjr.260423.cnv

**Published:** 2023-09-14

**Authors:** Aarthi Rajendran, Debashis Maikap, Prasanta Padhan, Ramnath Misra, Pratima Singh

**Affiliations:** 1 Department of Pulmonary Medicine, Kalinga Institute of Medical Sciences, KIIT University, Bhubaneswar, Odisha, India; 2 Department of Clinical Immunology and Rheumatology, Kalinga Institute of Medical Sciences, KIIT University, Bhubaneswar, Odisha, India

**Keywords:** nailfold capillaroscopic patterns, mixed connective tissue disorder, systemic sclerosis, interstitial lung disease, forced vital capacity

## Abstract

**Objective:**

To differentiate the nailfold capillaroscopy (NFC) findings in patients with MCTD-ILD and SSc-ILD and correlate the NFC changes and lung functions among them.

**Methods:**

In this observational study from Oct 2020 to Oct 2022, 27 patients with MCTD-ILD and 27 patients with SSc-ILD were included. NFC was performed using Jiangsu Jiahua, JH 1004, China. Statistical analysis was conducted using IBM SPSS software, version 26, and tests including Mann-Whitney U-test, student t-test, chi-square test, or Fisher’s exact test were used to compare between groups.

**Results:**

In this study, major capillaroscopic changes were more frequent in SSc-ILD group (92%) than in MCTD-ILD group (72.3%), with normal capillaries seen in 7.4% of MCTD-ILD cases. The mean FVC was higher in SSc-ILD group compared to MCTD-ILD group, and patients with capillary loss had a lower mean FVC. Loss of capillaries was more frequent in SSc-ILD group, while dilated capillaries were predominantly observed in MCTD-ILD group. A significant association was found between the severity of restriction in spirometry and NFC.

**Conclusion:**

There is an important role for NFC in detecting the severity of lung involvement, as the grading of restrictive severity in spirometry is strongly associated with capillaroscopic abnormalities.

## INTRODUCTION

“Interstitial lung disease” is defined as a heterogeneous group of diffuse parenchymal infiltrative disorders that impair the gas exchange function of the lung by disrupting the alveolar walls. They comprise a varying aetiology with some similarities in clinical, imaging, physiological, and pathological features.^1^ Connective tissue disease associated with interstitial lung disease (CTD-ILD) is defined as evidence of ILD demonstrated by computed tomography (CT), such as some combination of reticulation, ground-glass opacities, traction bronchiectasis, honeycombing, and/or cysts, in the setting of an established CTD. The clinical manifestations of these diseases result from inflammatory infiltration of the interstitium and capillary endothelium. The key element in the pathogenesis of this disease is the dysfunction of endothelial cells and fibroblasts, as well as abnormalities of the microvascular system that lead to hypoxia in tissues and altered immune responses.^2^

Nailfold capillaroscopy (NFC) is an established method for identifying microvascular abnormalities. It helps visualise changes in the nailfold capillary among various CTDs^3^ and is the best non-invasive method for examining and diagnosing microvascular abnormal patterns in secondary Raynaud’s phenomenon (RP) patients. As a result, early diagnosis of CTDs such as systemic sclerosis is possible.^4^ It has been suggested that NFC is involved in the study of Digital Ulcers, Scleroderma Renal Crisis, and PAH in systemic sclerosis.^5^ Interstitial lung disease (ILD) and pulmonary artery hypertension (PAH) are the main causes of disease-related mortality associated with SSc. However, there is a paucity of data regarding NFC’s role in predicting ILD and its association with the extent of ILD and changes in systemic sclerosis.^2^ NFC findings and internal organ involvement in MCTD patients have only been examined in one study so far in the literature.^3^ To date, no studies have compared NFC findings in MCTDILD vs SSc-ILD.

## OBJECTIVE

To differentiate the nailfold video capillaroscopy (NVC) findings in patients with MCTD-ILD and SSc- ILD and to correlate the NVC findings and lung functions in patients with MCTD-ILD and SSc-ILD.

## MATERIALS AND METHODS

This cross-sectional observational study was conducted in the Departments of Respiratory Medicine and Clinical Immunology and Rheumatology at Kalinga Institute of Medical Sciences, Bhubaneswar. Fifty-four consecutive patients, of which 27 were diagnosed with MCTD-ILD and 27 with SSc-ILD, were included in the study from October 2020 to October 2022.

Patients with chronic illnesses such as cirrhosis, chronic kidney disease, congestive cardiac failure, HIV and hepatitis B and C, peripheral vascular diseases, critically ill patients admitted to the intensive care unit, and patients with other forms of connective tissue disorder except for MCTD and SSc were excluded from the study. Informed consent was obtained from all patients and the study was approved by the Institutional Ethics Committee (Study approval number: KIIT/KIMS/IEC/428/2020)

The proforma included questionnaires on patients’ medical history, comorbidities, spirometry, six-minute walk test, pulse oximetry (SpO2), 2D echocardiogram, NVC, high-resolution computed tomography (HRCT), and further laboratory tests. Cardiologists performed 2D echocardiography using the Philips Imaging System epiq-7. Spirometry was performed using WinspiroPRO 8.1 software and a computer-based spirometer made by MIR Minister. PFT was classified into restrictive, obstructive, and mixed patterns.^6^ Further severity classification was done using the same BTS guidelines. Nail-Fold video capillaroscopy was performed using Jiangsu Jiahua, JH 1004, China.

Nailfold capillaroscopy was performed in a room temperature and examined with the help of a digital microscope at 200x magnification. A few drops of immersion oil were placed over the nailfold capillary bed to increase the keratin layer’s translucency. It was performed on all fingers except for the thumbs, and images were captured and stored in a computer for further analysis. To measure the correct density and dimension of capillaries, these images were analysed with the device’s software.

Interstitial lung diseases (ILDs) are defined by diffuse parenchymal infiltration of lung tissues, which are classified based on their aetiology, clinical presentation, and radiological findings.^7^ On HRCT thorax, ILD is classified in 3 stages: a few ground-glass or fibro reticular opacities in the lower lobes of the HRCT are indicative of early ILD, along with mild sub-pleural reticulation. Ground glass opacities (GGOs) along with reticulo-nodular interstitial thickening are present in peripheral lung fields bilaterally in the active stage of ILD. Extensive sub-pleural reticulation, ground-glass opacities, with honeycombing are characteristics of late stage ILD.^8^

For diagnosing systemic sclerosis, new criteria published by ACR and EULAR classification^9^ were used, which include thickening of the skin of the fingers, digital tip ulcers, Raynaud’s phenomenon, telangiectasia, NFC abnormalities, pulmonary hypertension, and/or ILD, and SSc-linked autoantibodies such as anti-topoisomerase I, anti-centromere, and anti-RNA polymerase III. The diagnostic criteria used for MCTD include the following three: Alarcón-Segovia,^10^ which includes serological criteria as positive titre of U1 RNP antibodies > 1:1600 and clinical criteria that include oedema of the hands, synovitis, myositis, Raynaud’s phenomenon, and acrosclerosis.

In our study, pulmonary hypertension was defined by PASP > 35mmHg in 2D echocardiogram by TR peak velocity method.^11^ A value greater than or equal to 35 mmHg is considered PAH and classified as follows: mild PAH (35–50 mmHg), moderate PAH (50–70 mmHg) and severe PAH (>70mmHg).^12^

Capillaroscopic patterns are defined based on the severity of abnormalities as follows:

1. Normal pattern shows 7–10 capillaries/mm with hairpin-shaped loops arranged in parallel rows and absence of microhaemorrhages and abnormal shapes such as tortuous loops or crosses.
2. Minor abnormalities show 7–10 capillaries/mm with less than 50% tortuous loops and hairpin-shaped loops arranged in parallel rows with no presence of microhaemorrhages or neoangiogenesis.
3. Major abnormalities are characterized by a reduction in capillary density, more than 50% tortuous loops, enlarged capillaries, abnormal shapes/disarranged loops with microhaemorrhages, more than 50% neoangiogenesis, and ectasia.^13^

**Figure 1.**
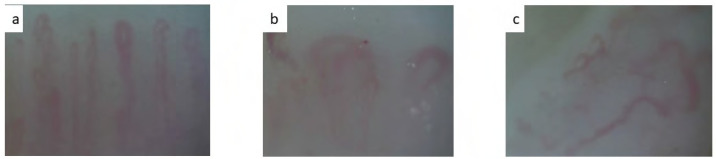
Nailfold capillaroscopy showed: **(a)** early scleroderma pattern, **(b)** active scleroderma pattern, **(c)** late scleroderma pattern.

In 2000, Cutolo et al. had classified progressive changes in microangiopathy as follows, Early pattern has few enlarged capillaries with organised capillary structure with preserved architecture and no evidence of capillary loss; active pattern has frequent enlarged or giant capillaries, absent or mild ramified capillaries with moderate capillary loss and mild disorganisation of capillary array; and late pattern has extensive avascular areas with abnormal shapes or ramified/bushy capillaries and severe architectural distortion with absence of giant capillaries and microhemorrhages.^14^

In our study we have differentiated both groups with both capillaroscopic abnormalities and patterns based on Cutolo et al. study. Regarding treatment, both the groups were treated with anti-fibrotics and immunosuppressants. Steroids were given as 0.5 to 1mg/kg in MCTD-ILD group and less than 10mg/day for SSc-ILD group.

### Statistical analysis

IBM SPSS software, version 26, was used to carry out the statistical analysis. The continuous variables in this study were presented as the mean with standard deviation. The significance for FVC was calculated using a Student’s t-test. The Mann-Whitney U-test was used to calculate the p-value for other variables. Depending on the context, either the Chi-square test or Fisher’s exact test was used to compare nominal categorical variables between the groups. The results were deemed statistically significant if the p-value was less than 0.05.

## RESULTS

Clinical features and demographic parameters among patients of both groups were depicted in **Table 1**. Among the 54 consecutive cases, the overall mean age was 43.80 ± 12.40. The mean age of the MCTD-ILD group was 39.96 ± 12.45, whereas in SSc-ILD, the mean age was 47.63 ± 11.32. There was a significant female preponderance with M:F = 1:5 (16.7% vs 83.3%) (p=0.001). Among the MCTD-ILD group, there were 81% females and 19% males, and in the SSc-ILD group, there were 85% females and 15% males.

**Table 1.** Demographic and clinical parameters.

**Demographics**	**MCTD-ILD (n=27)**	**SSc-ILD (n=27)**	**P Value**
Age in Years, Mean ± SD	39.96 ± 12.45	47.63 ± 11.32	
Gender, n (%)			
Male	5 (19%)	4 (15%)	0.715
Female	22 (81%)	23 (85%)	
Duration of the disease in Years, mean ± SD	4 ± 5	5 ± 4	
**Clinical Features, n (%)**			
Dyspnoea	20 (74.07%)	23 (85.19%)	0.310
Dry Cough	14 (51.85%)	21 (77.78%)	0.040
Reflux symptoms	12 (44.44%)	20 (74.07%)	0.020
Polyarthralgia	27 (100%)	22 (81.48%)	0.010
Chest Pain	6 (22.22%)	7 (25.93%)	0.750
Raynaud’s phenomenon	20 (74.07%)	25 (92.59%)	0.060
Sclerodactyly	16 (59.26%)	26 (96.3%)	0.001
Telangiectasia	2 (7.41%)	0 (0%)	0.490
Clubbing	0 (0%)	6 (22.22%)	0.020
**Spirometry**, **Mean** ± **SD**			
FEV1 (L)	2.37 ± 3.14	1.30 ± 0.52	0.001
FEV1 (%)	69.24 ± 18.26	54.50 ± 18.11	0.007
FVC (L)	1.84 ± 0.44	1.48 ± 0.52	0.010
FVC (%)	64.81 ± 15.11	52.25 ± 15.22	0.005
FEV1/FVC (%)	104.04 ± 22.84	101 ± 22.54	0.001
Mean 6MW distance covered (in mts)	331 ± 67.7	304.52 ± 93.72	0.020
Baseline SpO2 (%)	97.7 ± 1.49	94.81 ± 3.82	0.001
Post SpO2 (%), n (%)	94.85 ± 4.01	89.40 ± 8.46	0.001
**HRCT Findings, n (%)**			
NSIP	21 (77.78%)	19 (70.37%)	0.535
UIP	6 (22.22%)	8 (29.63%)	
**ILD Pattern, n (%)**			0.150
Early	15 (55.56%)	8 (29.63%)	
Active	7 (25.93%)	11 (40.74%)	
Late	5 (18.52%)	8 (29.63%)	
**PAH, n (%)**			
Normal	22 (81.48%)	17 (62.96%)	0.270
Mild	5 (18.52%)	8 (29.63%)	
Moderate	0 (0%)	1 (3.7%)	
Severe	0 (0%)	1 (3.7%)	

On comparing symptoms and signs, polyarthralgia was the most common symptom among MCTD-ILD patients seen in all cases, whereas sclerodactyly was the most common finding in SSc-ILD (96.3%) as compared to MCTD-ILD (59.26%). Dry cough was seen in 51.85% patients with MCTD-ILD and 77.78% patients with SSc-ILD, respectively. Reflux symptoms were reported by 44.44% vs 74.07% patients with MCTD-ILD and SSc-ILD, respectively (p=0.01). Dyspnoea was seen in 74.07% vs 85.19% and Raynaud’s phenomenon in 74.07% vs 92.59% among MCTD-ILD and SSc-ILD, respectively. Clubbing was seen only in SSc-ILD in 22.2% of cases. The maximum comorbid condition seen in our study was GERD followed by hypothyroidism, hypertension, and T2DM. GERD was seen in 44.44% patients with MCTD-ILD and 74.07% patients with SSc-ILD, respectively (p=0.02).

All patients underwent spirometric analysis, except for 3 patients who could not perform the test due to baseline grade-4 dyspnoea or oxygen therapy, and all belonged to the SSc-ILD group. The majority of cases (87%) had a restrictive pattern, and 1 patient had a mixed pattern belonging to the SSc-ILD group, while 3 patients had normal spirometry belonging to the MCTD-ILD group.

The grading of severity for restriction was mild (22.2% vs. 12.5%), moderate (40.7% vs. 20.8%), moderately severe (14.8% vs. 20.8%), severe (7.4% vs. 33.3%), and very severe (3.7% vs. 12.5%) cases in MCTD-ILD and SSc-ILD, respectively. The overall mean FEV1 was 1.86 L and 62.30%, mean FVC was 1.67 L and 58.90%, and FEV1/FVC (%) was 102.85, which clearly shows the predominance of the restrictive pattern in our study. Three of the 54 patients could not complete the six-minute walk test because their baseline SpO2 was less than 85% or they had grade 4 baseline dyspnoea. Seven people were unable to finish the test due to severe dyspnoea or lower limb pain. A six-minute walk test was completed by 44 patients.

HRCT thorax findings suggestive of NSIP pattern were seen in 77.78% and 70.37%, respectively, whereas UIP pattern was seen in 22.22% and 29.63% of MCTD-ILD and SSc-ILD groups, respectively. Regarding the pattern of severity findings in HRCT thorax, the majority of cases (55.56%) showed early changes of ILD in MCTD-ILD, whereas in SSc-ILD, 40.74% cases were found to be among the active pattern, and 29% cases showed late changes.

Among the 2 groups of MCTD-ILD and SSc-ILD, taking SSc-ILD as reference category, it was found that MCTDILD showed lesser late pattern of NFC from multivariable regression analysis (**Table 2**). It was seen that the duration of disease was more in late pattern of NFC. It was seen that the 6-minute walk distance was lesser in active pattern of NFC.

**Table 2.** Multiple logistic regression for NFC outcomes.

**NFC PATTERN**	**Parameters**	**Odds Ratio (OR)**	**95% CI for OR**		**P Value**
			**Lower Bound**	**Upper Bound**	
ACTIVE	AGE	0.975	0.9	1.056	0.533
	SEX [=Male]	0.23	0.02	2.648	0.239
	DURATION (IN YEARS)	1.119	0.8	1.566	0.512
	DISTANCE	0.985	0.973	0.998	0.020
	FVC (%)	1.001	0.942	1.065	0.962
	RANYAUDS PHENOMENON [=Present]	0.26	0.024	2.851	0.270
	PAH [=PRESENT]	3.453	0.257	46.448	0.350
	DIAGNOSIS [=MCTD-ILD]	0.965	0.109	8.57	0.975
LATE	AGE (IN YEARS)	0.977	0.891	1.072	0.624
	SEX [=Male]	0.565	0.04	7.994	0.672
	DURATION (IN YEARS)	1.566	1.066	2.3	0.022
	DISTANCE	1	0.987	1.013	0.986
	FVC (%)	1.01	0.943	1.082	0.770
	RANYAUD’S PHENOMENON [=Present]	2.365	0.091	61.387	0.604
	PAH [=PRESENT]	0.292	0.015	5.765	0.418
	DIAGNOSIS [=MCTD-ILD]	0.068	0.005	0.949	0.046

Reference category early.

### NFC Findings

The disease duration for all cases was 4 ± 7 years. In MTCD-ILD, 4 ± 5 years and in SSc-ILD, 5 ± 4 years. There was not much difference in each group in our study. The mean number of capillaries per millimetre was 4.26 ± 2.28. Among the MCTD-ILD group, the mean number of capillaries per millimetre was 4.8 ± 2.0, while among the SSc-ILD group it was 3.7 ± 1.9 (p=0.04). **Table 3** showed the differences in the morphology of various capillaroscopic parameters between the two groups. We analysed six variables, including loss of capillaries or avascular areas, dilated, or enlarged capillaries, giant capillaries, microhaemorrhages, ramification or abnormal shapes, and loss of architecture of the capillaries of the nail bed, to establish a comparative study. The NFC findings in the MCTD-ILD group showed that the majority of patients had dilated capillaries (96.3%), followed by loss of capillaries (70.4%), architectural disorganisation (48.1%), giant capillaries (37%), micro-haemorrhages (29.6%), and abnormal shapes or ramification (least frequent). In the SSc-ILD group, the major NFC abnormality was capillary loss (96.2%), followed by architectural disorganisation (74.1%), dilated capillaries (70.4%), giant capillaries (44.4%), microhaemorrhages (33.3%), and ramification (25.9%).

**Table 3.** Associations of capillaroscopic abnormalities and NFC pattern between MCTD-ILD and SSc-ILD.

**Capillaroscopic abnormalities**	**MCTD-ILD (n=27)**	**SSc-ILD (n=27)**	**P Value**
No. of capillaries/field (mm)	4.8 ± 2.0	3.7 ± 1.9	0.040
Capillary loss	19 (70.4%)	26 (96.2%)	0.020
Dilated Capillaries	26 (96.3%)	19 (70.4%)	0.020
Giant Capillaries	13 (48.1%)	12 (44.4%)	0.780
Microhemorrhages	8 (29.6%)	9 (33.3%)	0.770
Ramification	10 (37%)	7 (25.9%)	0.370
Architectural Loss	18 (66.7%)	20 (74.1%)	0.550
**NFC abnormality pattern of severity**			
Normal	2 (7.4%)	0 (0%)	0.070
Minor findings	5 (18.5%)	2 (7.4%)	0.100
Major findings	20 (74%)	25 (92%)	0.030
**NFC patterns based on Cutolo criteria**			
Early	8(29.6%)	4(14.8%)	0.090
Active	14(51.9%)	11(40.7%)	0.200
Late	5(18.5%)	12(44.4%)	0.020

**Table 3** showed a comparison of the nailfold pattern of severity between the MCTD-ILD and SSc-ILD groups. Most of the patients with major abnormalities (92%) were in the SSc-ILD group, which was statistically significant (p=0.03). 18.5% of patients with MCTD-ILD had minor capillary changes, while only 7.4% of patients with SSc-ILD had minor capillary changes.

PAH was observed in 29.6% of cases, with the majority being from the SSc-ILD group. Of these, 15 cases showed major changes, but no significant correlation was observed between PAH and NFC changes (p=1.81). We compared the pattern of severity of restriction in spirometry with the nailfold capillaroscopic changes, and found a significant association (p=0.03) (**Table 4**). The mean FVC was 64.81 ± 15.11 vs 52.25 ± 15.22, lower in the SSc-ILD group. Mean FVC was 56.74 ± 15.52 among those with loss of capillaries, while it was 69 ± 16.91 among those without capillary loss (p=0.03), indicating a significant correlation between FVC (%) and loss of capillaries in our study.

**Table 4.** Comparison of pattern of severity in spirometry with capillaroscopic change in MCTD-ILD and SSc-ILD groups.

**Spirometry pattern of severity**	**Normal NFC (n=2)**	**Minor NFC changes (n=6)**	**Major NFC changes (n=43)**	**p-value**
Normal	1 (33.3%)	1 (33.3%)	1 (33.3%)	0.03
Mild	1 (11.1%)	2 (22.2%)	6 (66.7%)	
Moderate	0	1 (6.3%)	15 (93.8%)	
Moderate-severe	0	0	9 (100%)	
Severe	0	2 (20%)	8 (80%)	
Very severe	0	0	4 (100%)	

## DISCUSSION

We conducted a detailed nailfold capillaroscopic analysis to compare the NFC patterns between MCTDILD and SSc-ILD groups of patients. The number of capillaries in the SSc-ILD group was significantly lower than that in the MCTD-ILD group. Previous studies by Kramer et al.^15^ and Paolino et al.^4^ have shown a higher mean capillary density in MCTD-ILD (7.6 ± 1.6) and SSc-ILD (4.9 ± 1.6), respectively, compared to our study. These findings may be attributed to several pathophysiological disease mechanisms that cause progressive systemic vascular dysfunction. In our study, 70.4% of MCTD-ILD patients had loss of capillaries, while 96% of SSc-ILD patients showed the same. Studies have shown that capillary loss/avascular areas are more common in SSc patients with pulmonary, cardiac, and cutaneous manifestations.^16–18^

Using capillaroscopy as a non-invasive method could potentially predict the involvement and progression of CTD-ILD.^19^ Similarly, in a study by de Holanda Diogenes et al., MCTD patients with ILD had more capillary loss than patients without ILD.^20^ Avascularisation areas significantly correlate with a higher prevalence of lung fibrosis, which was given in a study by Niklas K.^21^

Capillary loss and architectural distortion were seen more prominently in SSc-ILD than MCTD-ILD. There are no comparative studies associated with ILD; however, there are single reports depicting the relation between SSc and MCTD capillaroscopy findings, which suggest that abnormalities in capillaries are less frequent in MCTD than in SSc, similar to our study.^20^

Nevertheless, the presence of dilated/giant capillaries and ramification was slightly higher in numbers in MCTDILD in our study. Therefore, capillaroscopic changes in them may correlate with disease progression and organ involvement, similar to systemic sclerosis patients. In a study by Lowenhoff et al., giant capillaries were seen in 83% of cases of MCTD with ILD compared to zero cases without ILD, stating that enlarged/giant capillaries can serve as an early marker in lung involvement in MCTD. It is believed that giant capillaries are due to a local auto-regulatory response to tissue hypoxia; the dilation may appear as the initial sign of capillary wall disruption.^3^ In our study, most of the patients with major capillary abnormalities (92%) were in the SSc-ILD group. All the patients with a normal NFC pattern belonged to the MCTD-ILD group. 18.5% of patients with MCTD-ILD had minor capillary changes, whereas only 7.4% of patients with SSc-ILD had minor capillary changes. There is only one study that compared NFC changes between SSc and MCTD, which is a study by Paolino et al.^4^ This study stated that nailfold capillary damage in MCTD patients is less progressive than in systemic sclerosis patients, similar to our study. However, there is no previous study comparing MCTD and SSc associated with ILD as we have done in this study.

All patients with PAH had abnormal capillaroscopic findings in a study by Markusse et al., which is similar to our study. 15 out of 16 cases of PAH had major capillaroscopic changes, stating that severe nailfold capillaroscopic changes are associated with ILD and PAH in SSc in our study.^22^ In a study by Todoroki et al., nailfold capillaroscopic patterns were observed in all MCTD patients with PAH, and in 21.0% of those without PAH which was not in concordance with our study were 83.0 % of MCTD-ILD had no PAH. ^23^

On comparing the mean FVC and 6MWT with nailfold capillaroscopic changes respectively, no correlation was observed in our study. However, Caramaschi et al.^24^ found a strong association between FVC, DLCO values, and NFC patterns. Nevertheless, our findings indicated a significant correlation of FVC (%) with loss of capillaries. Our results were partially consistent with the reports of Corrado et al., who found that loss of capillary density was present in patients with ILD in comparison to those without ILD and was also more pronounced in SSc associated with ILD, pointing towards the fact that NFC plays an important role in detecting lung involvement.^25^

In ILD, pulmonary function abnormalities usually reflect a restrictive lung defect with reduced lung compliance and lung volumes. The decline in FVC has clinically significant association with the risk of SSc-related hospitalisation in patients with SSc-ILD reported in the SENSCIS trial. Hence, serial FVC monitoring is important for assessing the SSc-ILD patients in terms of preventing hospitalisation and early death.^26^

All the cases belonging to the very severe restrictive pattern and 80% of cases with severe restrictive pattern, and all cases showing moderate-severe restrictive pattern have major NFC abnormalities, suggesting that higher severity of pulmonary lung function is directly proportional to capillaroscopic changes. So far, there were no studies showing correlation between NFC changes and grading of severity pattern in PFT. Moreover, in our study, among SSc-ILD, a greater number of patients belonged to severe and very severe restrictive pattern compared to MCTD-ILD.

In our study, we found that even in normal spirometry, NFC changes were observed in MCTD-ILD patients, whereas a study by Cutolo M et al.^27^ mentioned that NFC changes might appear even before systemic manifestations appear in SSc. Hence, NFC can serve as a technique in the early detection of lung involvement in MCTD patients as well. The disease’s most important clinical characteristic is microvascular damage. Therefore, the appropriate instrument for investigating structural microvasculopathy is NFC.^28^

To the best of our knowledge, this is the first study that compared nailfold capillaroscopic abnormalities among MCTD-ILD and SSc-ILD. We found a correlation between NFC changes and grading of severity pattern in spirometry. NFC can also be used for early detection of lung involvement in MCTD patients despite a normal spirometric pattern. The limitation of our study includes a small sample size in each group. Our study design lacked follow-up, which might have helped in differentiating the major patterns among both groups. The disease duration at the time of NFC analysis and the ongoing treatment might interfere with microvascular disease progression and the results.

## CONCLUSION

Major capillaroscopic changes were observed in a majority of SSc-ILD cases, indicating that nailfold capillary damage in MCTD-ILD patients is less progressive than in systemic sclerosis-ILD patients. Capillary loss was more predominant in SSc-ILD, while dilated/enlarged capillaries were common in MCTD-ILD. The severity grading of pulmonary function was strongly associated with capillaroscopic abnormalities, and a significant correlation was found with forced vital capacity (FVC) and loss of capillaries, emphasising the important role of NFC in detecting the severity of lung involvement. NFC can be used as a basic screening tool for assessing disease severity in patients with SSc and MCTD associated with ILD, along with other pulmonary function tests.

## ABBREVIATIONS:

CTD-ILD: Connective tissue disorder associated with interstitial lung diseases
FEV1: Forced expiratory volume in first second
FVC: Forced vital capacity
MCTD-ILD: Mixed connective tissue disease associated interstitial lung disease
NFC: Nailfold capillaroscopy
NVC: Nailfold video capillaroscopy
SSc-ILD: Systemic sclerosis associated interstitial lung disease
